# Supplementary material for: Synthesis of substituted biphenyls and in vitro evaluation of antimicrobial and anti-biofilm activities supported by in silico ADMET prediction
Source: PLoS One. 2026 Apr 6;21(4):e0346142. doi: 10.1371/journal.pone.0346142 (PMC13052900; doi:10.1371/journal.pone.0346142)
Supplement: S1 File — (DOCX) [file pone.0346142.s001.docx]

**Synthesis of Substituted Biphenyls and In Vitro Evaluation of Antimicrobial and Anti-Biofilm Activities Supported by In Silico ADMET Prediction**

**Saad Alghamdi^1^, Ahmed Hassen Shntaif^2*^,** **Yasser Hussein Issa Mohammed^3^**^*^

^1^Department of Clinical Laboratory Sciences, Faculty of Applied Medical Sciences, Umm Al-Qura University, Makkah, Saudi Arabia. https://orcid.org/0000-0003-4532-9128

^2^Department of Chemistry, College of Science for Women, University of Babylon, Alhilla, Iraq. <https://orcid.org/0000-0003-0723-5622>

^3^Department of Pharmacy, Collage of Medicine and Health Science, Hajjah University, Hajjah, Yemen. <https://orcid.org/0000-0003-1086-7292>

* Corresponding authors

E-mail: [wsc.ahmed.hassan@uobabylon.edu.iq](mailto:wsc.ahmed.hassan@uobabylon.edu.iq)

[issayasser16@gmail.com](mailto:issayasser16@gmail.com)


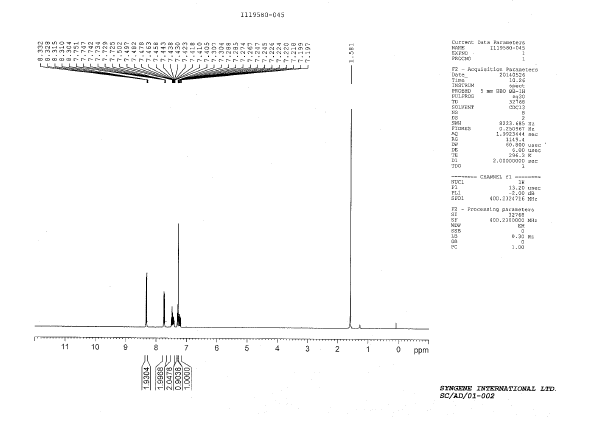


Fig. 1S: HNMR for compound 3a


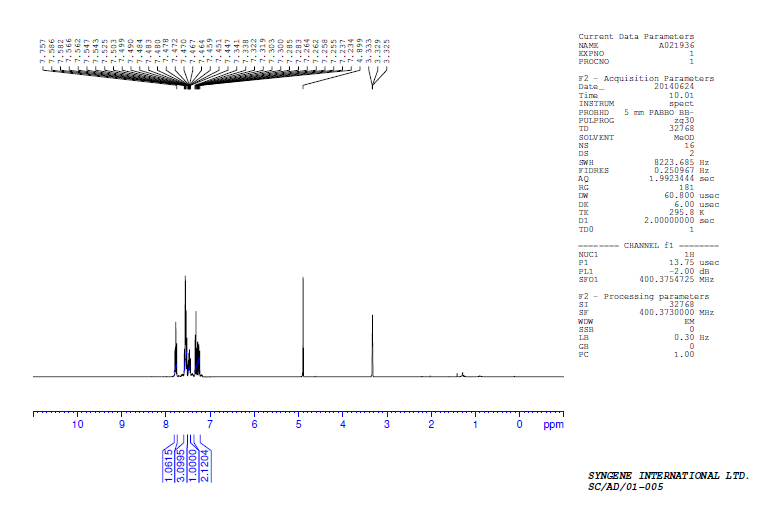


Fig. 2S: HNMR for compound 3b


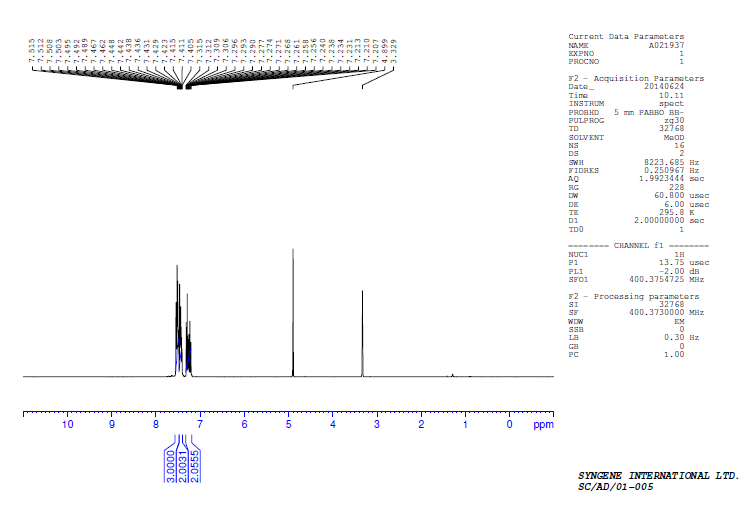


Fig. 3S: HNMR for compound 3c


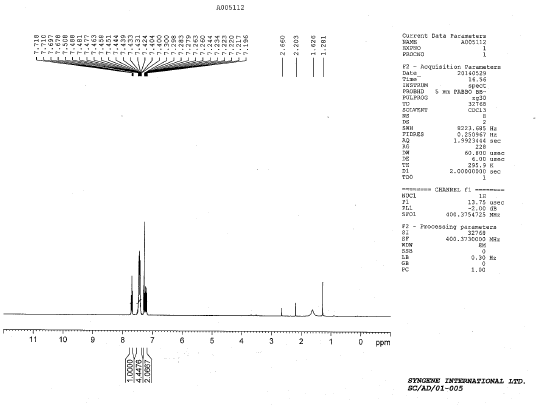


Fig. 4S: HNMR for compound 3d


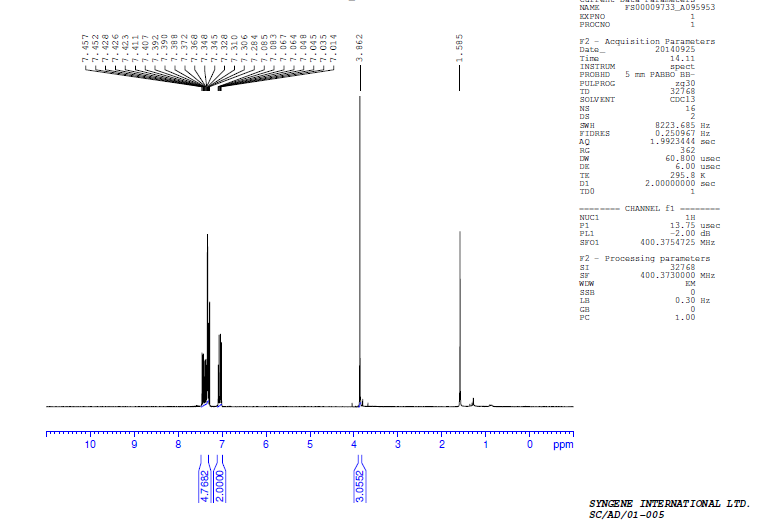


Fig. 5S: HNMR for compound 3e


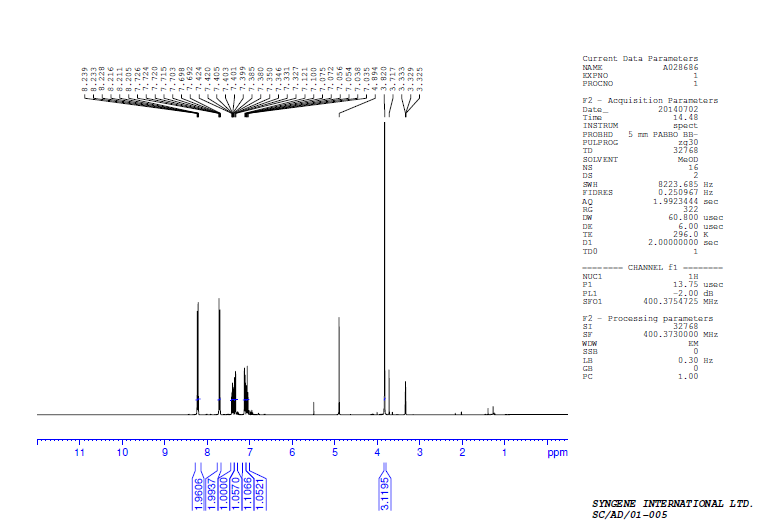


Fig. 6S: HNMR for compound 3f


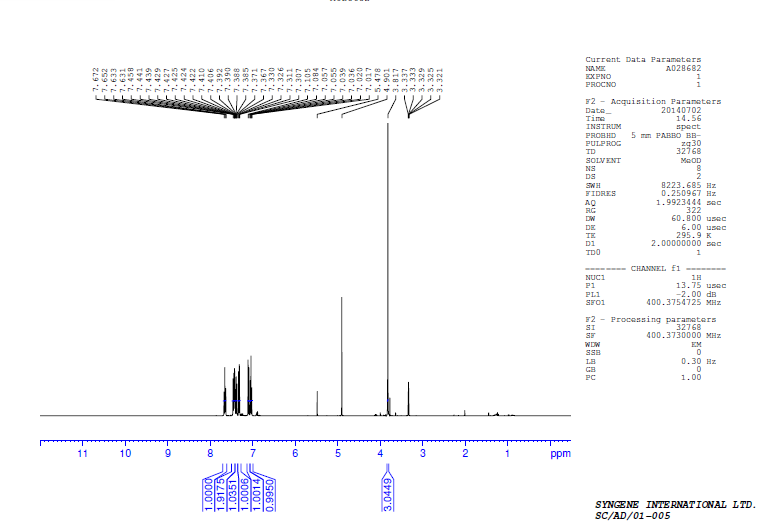


Fig. 7S: HNMR for compound 3g
