# Supplementary material for: Synthesis of substituted biphenyls and in vitro evaluation of antimicrobial and anti-biofilm activities supported by in silico ADMET prediction
Source: PLoS One. 2026 Apr 6;21(4):e0346142. doi: 10.1371/journal.pone.0346142 (PMC13052900; doi:10.1371/journal.pone.0346142)
Supplement: Attachment — Submitted filename: reviewer #.docx [file pone.0346142.s002.docx]

The manuscript “Revolutionizing Therapeutics: Synthesizing Substituted Biphenyls and Assessing Their Antimicrobial Properties, ADMET Properties, and Biofilm Inhibition Potential” (Manuscript ID: PONE-D-25-65844). This manuscript describes the synthesis, in silico ADMET profiling, and in vitro antimicrobial/antibiofilm evaluation of a series of substituted biphenyl compounds. However, the manuscript suffers from significant methodological, reporting, and interpretive weaknesses that must be addressed before it can be considered for publication

**1. Critical Ethical and Compliance Deficiencies**

Human Subjects Research: The manuscript uses clinical isolates from urine specimens obtained from "the Microbiology laboratory at the College of Applied Sciences, University of Hajjh," but no ethical approval statement or IRB approval number is provided.

Informed Consent: There is no statement regarding informed consent from patients or waiver thereof. This is mandatory for publication.

**2.** **Fundamental Methodological Flaws in Biological Testing**

Non-Standard MIC Determination: Using agar well diffusion for MIC is not CLSI/EUCAST compliant. The gold standard is broth microdilution. Agar diffusion cannot accurately determine MIC values, especially for compounds with poor solubility.

The choice of three clinical isolates (only *S. aureus*, *E. coli*, and *C. albicans*) is very limited; there is no description of the number of independent isolates, their resistance profiles, or whether they represent clinically relevant multidrug-resistant strains

Inconsistent Methodology: The methods section mentions AutoDockTools-1.5.6 for docking, but the results describe MOE software. The procedure section states 65°C reaction temperature, while the results state 72°C. These contradictions undermine credibility.

Lack of Validation: No positive/negative controls were included for all compounds in all assays. DMSO concentration was not standardized across tests, introducing confounding variables.

**3. Inadequate Statistical Analysis and Reproducibility**

No Statistical Framework: The manuscript completely lacks statistical analysis. Values are presented as "mean ± SD" without stating the number of replicates (n), statistical tests used, or significance thresholds.

High Variability: The reported SD values are often >50% of the mean (e.g., Table 9: MIC for *S*. *aureus* = 7.13 ± 4.01 mg/mL), indicating poor experimental reproducibility that is not addressed.

**4. Incomplete and Inconsistent Chemical Characterization**

Missing Data: Compounds 3e-3k lack spectroscopic characterization (only melting points). No purity data (e.g., HPLC traces) is provided for any compound.

- Structural Errors: The abstract names compound 3i as "2-methoxy-4'-nitrophenyl" (missing "biphenyl"). **Table 1** lists formula C₁₃H₁₁NO₃, but this does not match the drawn structure in **Scheme 1**.
- Yields Unreported: No yields provided for compounds 3b-3e.

-	The title and abstract use strong language such as “Revolutionizing Therapeutics” and “promising antimicrobial agents capable of addressing antibiotic-resistant infections”, which is not fully justified by screening data in only three laboratory strains without in vivo validation or resistance profiling

**5. Fundamentally Flawed Data Presentation**

Unusable Tables: Tables 2, 5, and 8 are garbled with misplaced headings, broken formatting, and incoherent content. Table 2 appears to have merged columns without values.

Incorrect Units: Table 1 lists "log Kp (cm/s)" with values -5.12 cm/s—log units cannot have cm/s dimension. MIC values are reported in mg/mL (unusually high) but the resazurin assay uses μg/mL—discrepancies of 1000-fold.

Mislabeled Figures: **Figure 9** caption mentions compound **3f** but should be 3i. Figure legends are incomplete (e.g., **Figure** 2, 4, 10 lack sufficient detail).

**6. Unsupported Conclusions and Overstatement**

Grandiose Claims: The title "Revolutionizing Therapeutics" and claims of "addressing antibiotic-resistant infections" are not supported by limited in vitro data against three unspecified strains.

No Clinical Relevance: MIC values of 3.4-8.35 mg/mL (3400-8350 μg/mL) are extremely poor compared to clinical antibiotics (typically <10 μg/mL). This is not discussed as a limitation.

Misinterpretation of Docking: Streptomycin has a better docking score (-7.23) than 3i (-6.48), yet the text claims "most compounds showed good inhibition with more binding affinity." No correlation coefficient between docking scores and biological activity is presented (R² likely <0.3).

**7. Lack of Structure-Activity Relationship (SAR) Analysis**

The manuscript synthesizes 11 analogs but provides zero discussion of SAR. Why does the 4-nitro substituent **(3i**) outperform others? What is the effect of fluorination (3c, 3e, 3h)? No molecular basis is proposed.

**8. Inappropriate *In Silico* Methods**

Wrong Protein Target: E. coli FabH is a bacterial enzyme. Docking against this target cannot explain antifungal (*C. albicans*) activity. No rationale for target selection is provided.

No Validation: No re-docking of co-crystallized ligand to validate the docking protocol (required RMSD <2 Å). No comparison with known FabH inhibitors.

ADMET Over-reliance: SwissADME and similar tools have limited accuracy. No experimental validation of any ADMET parameter (e.g., solubility, permeability).

**9- Language Quality**

The manuscript contains numerous grammatical errors, awkward phrasing, and non-standard scientific English throughout (e.g., "the reaction's progress," "competent synthesis," "the purpose of the present study was" repeated).

**10- Outdated and Irrelevant References**

References #11 (ophthalmic solutions) and #12 (Hsp90 in cancer) are unrelated to the study. Many references are >10 years old; more recent literature on biphenyl antimicrobials and Suzuki coupling advances is omitted.

Scheme 1: Poorly formatted with cramped text, unclear substitution patterns, and illegible atom labels.

**11- Inconsistent Controls**

Fluconazole is used as a control for *C. albicans* but not mentioned in methods. Streptomycin is used inconsistently across assays.

Missing DLS/Polydispersity: For nanoparticle-like properties, DLS data would be relevant but not included.

Terminology: "Gram-negative fungus strain Candida albicans" is incorrect—Candida is a yeast (fungus), not classified as Gram-negative.

Units: Inconsistent use of mg/mL vs. μg/mL. Standard is μg/mL or μM.

**Recommendation**

Rejection with Major Revision. The manuscript addresses a relevant topic but requires substantial additional experimentation, ethical documentation, and data re-analysis to meet PLOS ONE's standards. The chemical synthesis is promising, but the biological evaluation is methodologically flawed and the conclusions are overstated.
