# Supplementary material for: Synthesis of substituted biphenyls and in vitro evaluation of antimicrobial and anti-biofilm activities supported by in silico ADMET prediction
Source: PLoS One. 2026 Apr 6;21(4):e0346142. doi: 10.1371/journal.pone.0346142 (PMC13052900; doi:10.1371/journal.pone.0346142)
Supplement: Attachment — Submitted filename: Response to Reviewers.docx [file pone.0346142.s003.docx]

**Answer comments Editor:**

*Dear Editor: Thank you for the guidance regarding NMR reporting. The compounds in this study were synthesized and characterized previously, and only the final ¹H NMR spectrum figures are currently available for several compounds. The original raw NMR data files (FID), acquisition files, and processing parameters are no longer accessible due to the age of the experiments and data archiving limitations.*

*To comply as fully as possible with PLOS ONE requirements, we have provided the available full, clearly labeled ¹H NMR spectra with compound identifiers as Supporting Information. Where full characterization for specific compounds has been published previously, we cite the original publication in which complete spectral assignments are reported. We also added all available instrument and acquisition details to the Methods section; where certain parameters were not recorded at the time (e.g., concentration or temperature), this is stated explicitly.*

**Answer comments reviewer 1:**

We sincerely thank Reviewer 1 for the detailed and constructive comments. We have carefully revised the manuscript to improve scientific rigor, consistency, clarity, and data presentation. Below we respond point-by-point.

1. Critical Ethical and Compliance Deficiencies
   Human Subjects Research: The manuscript uses clinical isolates from urine specimens obtained from "the Microbiology laboratory at the College of Applied Sciences, University of Hajjh," but no ethical approval statement or IRB approval number is provided.
   Informed Consent: There is no statement regarding informed consent from patients or waiver thereof. This is mandatory for publication.

***Answer:*** *Thank you for raising this important issue. We have revised the manuscript to include an Ethics and Informed Consent/Waiver statement.*

2. Fundamental Methodological Flaws in Biological Testing
   Non-Standard MIC Determination: Using agar well diffusion for MIC is not CLSI/EUCAST compliant. The gold standard is broth microdilution. Agar diffusion cannot accurately determine MIC values, especially for compounds with poor solubility.
   The choice of three clinical isolates (only S. aureus, E. coli, and C. albicans) is very limited; there is no description of the number of independent isolates, their resistance profiles, or whether they represent clinically relevant multidrug-resistant strains
   Inconsistent Methodology: The methods section mentions AutoDockTools-1.5.6 for docking, but the results describe MOE software. The procedure section states 65°C reaction temperature, while the results state 72°C. These contradictions undermine credibility.
   Lack of Validation: No positive/negative controls were included for all compounds in all assays. DMSO concentration was not standardized across tests, introducing confounding variables.

***Answer:*** *Thank you for these detailed methodological comments. We agree that several sections required clarification and correction to ensure reproducibility and compliance with standard practices. We have revised the manuscript as follows:*

*2.1 Non-standard MIC determination (agar diffusion vs broth microdilution)*

*We agree that agar well diffusion is not CLSI/EUCAST compliant for MIC determination and that broth microdilution is the reference method. In the revised manuscript, we have removed the term “MIC” from the agar well diffusion assay and now report this endpoint as a diffusion-based inhibitory concentration (MIC_agar) or lowest inhibitory concentration in agar, which is used for comparative screening only. We further clarified that MIC_agar values are diffusion- and solubility-dependent and should not be interpreted as equivalent to MIC values obtained by broth microdilution.*

*To address standardization, we expanded the broth microdilution MIC method (resazurin-based microdilution) and cited standard susceptibility testing guidelines (e.g., CLSI M07 / ISO 20776-1; and EUCAST guidance where applicable).*

*2.2 Limited isolate panel and missing resistance profiling*

*We agree that testing only three organisms is limited. In the revised manuscript, we clarified that the strains were urine-derived clinical isolates provided by the Microbiology Laboratory and that assays were performed using these isolates as a preliminary screening panel. We also added an explicit limitation statement noting that resistance profiling (e.g., MRSA/ESBL) was not performed within the scope of this study and that future work will include a broader set of well-characterized multidrug-resistant isolates.*

*We also clarified the number of isolates tested and whether a single isolate per species or multiple independent isolates were used.*

*2.3 Inconsistent methodology statements (AutoDockTools vs MOE; 65 °C vs 72 °C)*

*We agree that these inconsistencies undermine credibility.*

*Additional proofreading was performed to ensure consistency of experimental conditions across all sections.*

*Change made in manuscript: Corrected docking software and reaction temperature statements across Methods/Results and figure captions.*

*2.4 Controls and DMSO standardization*

*We agree that appropriate controls and consistent solvent concentration are required. In the revised manuscript, we clearly stated and applied:*

*DMSO negative control at the same final concentration as in test solutions for every assay.*

*Streptomycin as positive control for bacterial assays and fluconazole as positive control for Candida assays.*

*All assays were performed in triplicate (n = 3) with independently prepared plates/wells, and results were reported as mean ± SD.*

3. Inadequate Statistical Analysis and Reproducibility
   No Statistical Framework: The manuscript completely lacks statistical analysis. Values are presented as "mean ± SD" without stating the number of replicates (n), statistical tests used, or significance thresholds. High Variability: The reported SD values are often >50% of the mean (e.g., Table 9: MIC for S. aureus = 7.13 ± 4.01 mg/mL), indicating poor experimental reproducibility that is not addressed.

***Answer:*** *Thank you for this comment. We agree that the original manuscript did not clearly report the statistical framework. In the revised version, we have now explicitly stated the number of replicates (n = 3), the statistical tests used (one-way ANOVA with Tukey’s HSD post-hoc test), and the significance threshold (p < 0.05).*

*Regarding the reported high variability, we found that the SD value highlighted by the reviewer (e.g., Table 9: MIC for S. aureus = 7.13 ± 4.01 mg/mL) resulted from a calculation/typographical error in the SD reporting. This has been corrected, and we performed a comprehensive recheck of all SD calculations across tables to ensure accuracy. The corrected tables are included in the revised manuscript.*

4. Incomplete and Inconsistent Chemical Characterization
   Missing Data: Compounds 3e-3k lack spectroscopic characterization (only melting points). No purity data (e.g., HPLC traces) is provided for any compound.
   - Structural Errors: The abstract names compound 3i as "2-methoxy-4'-nitrophenyl" (missing "biphenyl"). Table 1 lists formula C₁₃H₁₁NO₃, but this does not match the drawn structure in Scheme 1.
   - Yields Unreported: No yields provided for compounds 3b-3e.
   - The title and abstract use strong language such as “Revolutionizing Therapeutics” and “promising antimicrobial agents capable of addressing antibiotic-resistant infections”, which is not fully justified by screening data in only three laboratory strains without in vivo validation or resistance profiling

***Answer:*** *Thank you for these important comments. We agree that the original manuscript required improved chemical characterization reporting, correction of structural naming/formula, inclusion of missing yields, and more cautious wording in the title/abstract. The manuscript has been revised as follows:*

*4.1 Missing spectroscopic characterization and purity data (3e–3k; HPLC)*

*We acknowledge that detailed spectroscopic characterization (e.g., H/C NMR, IR, HRMS) and purity data were not sufficiently presented for compounds 3e–3k in the submitted version. These compounds were previously synthesized and fully characterized in our earlier publication. In the revised manuscript, we have:*

*Added a clear statement that compounds 3e–3k were previously prepared and characterized,*

*Cited the original publication where the full spectral data are provided,*

*While HPLC purity traces were not available within the scope of this study, compound identity was confirmed by spectroscopic methods and melting point comparison with literature*

*4.2 Structural naming and formula corrections*

*We agree that the abstract wording was incomplete and have corrected the compound name to include the scaffold, i.e., “2-methoxy-4′-nitro-biphenyl (3i)” (or “2-methoxy-4′-nitrobiphenyl”). We also corrected inconsistencies between Table 1 and Scheme 1. Specifically, the molecular formula for compound 3i was rechecked and corrected to match the drawn structure, and all calculated properties were updated accordingly.*

*4.3 Missing yields (3b–3e)*

*We agree that yields must be reported. The isolated yields for compounds 3b–3e have now been added to the Experimental section and/or Table of compounds.*

*4.4 Overstated title/abstract claims*

*We agree that the previous wording was too strong given the study scope (in vitro screening on a limited panel without in vivo validation or resistance profiling). The title and abstract were revised to remove overstatements.*

5. Fundamentally Flawed Data Presentation Unusable Tables: Tables 2, 5, and 8 are garbled with misplaced headings, broken formatting, and incoherent content. Table 2 appears to have merged columns without values.
   Incorrect Units: Table 1 lists "log Kp (cm/s)" with values -5.12 cm/s—log units cannot have cm/s dimension. MIC values are reported in mg/mL (unusually high) but the resazurin assay uses μg/mL—discrepancies of 1000-fold. Mislabeled Figures: Figure 9 caption mentions compound 3f but should be 3i. Figure legends are incomplete (e.g., Figure 2, 4, 10 lack sufficient detail).

***Answer:*** *Thank you for these detailed comments. We agree that several tables/figures required correction and clearer reporting. The manuscript has been revised as follows:*

*5.1 Unusable tables (Tables 2, 5, and 8)*

*We agree that Tables 2, 5, and 8 contained formatting issues that reduced readability. In the revised manuscript, these tables were reformatted with corrected headings, consistent alignment, and coherent content presentation. In particular, Table 2 was rebuilt to eliminate merged/broken columns, and Table 8 was reformatted to display concentrations and inhibition values clearly.*

*5.2 Incorrect units (log Kp and MIC unit mismatch)*

*(a) log Kp: We agree with the reviewer that log values are dimensionless and should not be reported with physical units attached to the numerical value. log10(Kp), where Kp is expressed in cm/s (as provided by SwissADME). Thus, the numeric entries remain dimensionless, and the unit applies to Kp prior to logarithmic transformation.*

*(b) MIC units: We agree that inconsistent reporting of mg/mL versus µg/mL creates confusion. In the revised manuscript, MIC values and test concentrations were standardized to µg/mL throughout the Methods, Results, and all tables/figures. Any values previously reported in mg/mL were checked and corrected/conformed to the intended unit system, eliminating the 1000-fold discrepancy.*

*5.3 Mislabeled and incomplete figures*

*We agree that Figure captions and legends must be accurate and sufficiently detailed. The caption of Figure 9 was corrected to refer to compound 3i (not 3f).*

6. Unsupported Conclusions and Overstatement
   Grandiose Claims: The title "Revolutionizing Therapeutics" and claims of "addressing antibiotic-resistant infections" are not supported by limited in vitro data against three unspecified strains.

No Clinical Relevance: MIC values of 3.4-8.35 mg/mL (3400-8350 μg/mL) are extremely poor compared to clinical antibiotics (typically <10 μg/mL). This is not discussed as a limitation.
Misinterpretation of Docking: Streptomycin has a better docking score (-7.23) than 3i (-6.48), yet the text claims "most compounds showed good inhibition with more binding affinity." No correlation coefficient between docking scores and biological activity is presented (R² likely <0.3).

***Answer:*** *Thank you for these important comments. We agree that the initial version overstated the scope and clinical implications of the findings, and that the docking interpretation required correction. The manuscript has been revised to ensure that conclusions are consistent with the experimental evidence and study limitations.*

*6.1 Grandiose claims / scope mismatch*

*We agree that phrases implying clinical efficacy (e.g., “Revolutionizing Therapeutics,” “addressing antibiotic-resistant infections”) are not justified by the present study, which is limited to in vitro screening on a small panel and does not include in vivo validation or resistance profiling. We therefore revised the title, abstract, and conclusion to use cautious language (e.g., “in vitro antimicrobial and anti-biofilm evaluation,” “preliminary screening,” “lead compound for further optimization”) and removed statements implying treatment of resistant infections.*

*6.2 MIC values and clinical relevance*

*Thank you for highlighting this issue. We confirm that the MIC values were determined and recorded in µg/mL, but were mistakenly reported in the manuscript as mg/mL due to a unit conversion/typing error.*

*In addition, we revised the docking interpretation to avoid overstatement. We now clarify that streptomycin has a more favorable docking score than 3i, and docking results are used qualitatively to describe binding poses rather than to claim superior affinity. We also removed statements implying a strong correlation between docking score and biological activity.*

*6.3 Docking interpretation and correlation with biological activity*

*We agree that docking results must be interpreted cautiously. Streptomycin produced a more favorable docking score (−7.23) than compound 3i (−6.48), and the revised text now reflects this. We also removed the statement suggesting that “most compounds showed better binding affinity,” and clarified that docking was used to propose binding modes and qualitative interactions rather than to claim potency. A correlation analysis between docking scores and biological activity was not the focus of this work and is not sufficient with the current dataset; this is now stated as a limitation.*

7. Lack of Structure-Activity Relationship (SAR) Analysis
   The manuscript synthesizes 11 analogs but provides zero discussion of SAR. Why does the 4-nitro substituent (3i) outperform others? What is the effect of fluorination (3c, 3e, 3h)? No molecular basis is proposed.

***Answer:*** *Thank you for this comment. We agree that the initial manuscript did not adequately discuss structure–activity relationships (SAR). In the revised version, we added a dedicated SAR subsection to the Discussion. This section compares the activity trends across compounds 3a–3k and proposes a preliminary molecular rationale for (i) the enhanced activity of the nitro-substituted derivative 3i, and (ii) the variable effects of fluorination (e.g., 3c, 3e, 3h) on antimicrobial and anti-biofilm behavior. While these SAR conclusions remain preliminary (limited strain panel and in vitro screening), they provide mechanistic hypotheses to guide future optimization.*

8. Inappropriate In Silico Methods
   Wrong Protein Target: E. coli FabH is a bacterial enzyme. Docking against this target cannot explain antifungal (C. albicans) activity. No rationale for target selection is provided.
   No Validation: No re-docking of co-crystallized ligand to validate the docking protocol (required RMSD <2 Å). No comparison with known FabH inhibitors.
   ADMET Over-reliance: SwissADME and similar tools have limited accuracy. No experimental validation of any ADMET parameter (e.g., solubility, permeability).

***Answer:*** *Thank you for these important comments. We agree that FabH is a bacterial enzyme and docking to this target cannot explain antifungal activity against Candida albicans. The manuscript has been revised to clarify the scope and interpretation of the in silico work:*

*(1) Target selection and study aim*

*The primary aim of this study is to evaluate the synthesized biphenyl derivatives as antibacterial candidates, and molecular docking was included to provide supportive mechanistic hypotheses for the antibacterial activity only. C. albicans was included as an additional organism to provide supporting broad-spectrum screening and to explore whether the compounds show activity beyond bacteria; however, we do not claim that FabH docking explains antifungal outcomes. This has now been explicitly stated in the Methods and Discussion.*

*(2) Docking validation*

*We agree that docking validation is required. We have revised the Methods to include protocol validation by re-docking the co-crystallized ligand from PDB ID 5BNR and assessing pose reproduction using RMSD, with RMSD < 2.0 Å as the acceptance criterion. We also clarified that docking scores are interpreted qualitatively to describe binding poses/interactions.*

*(3) ADMET prediction limitations*

*We agree that SwissADME and related tools provide approximate predictions. The manuscript has been revised to treat ADMET results as preliminary supportive estimates rather than definitive properties, and we added a limitation statement indicating that experimental validation (e.g., solubility, permeability, cytotoxicity) is needed in future work.*

9. Language Quality The manuscript contains numerous grammatical errors, awkward phrasing, and non-standard scientific English throughout (e.g., "the reaction's progress," "competent synthesis," "the purpose of the present study was" repeated).

***Answer:*** *Thank you for this comment. We agree that the manuscript required significant language improvement.*

10. Outdated and Irrelevant References
    References #11 (ophthalmic solutions) and #12 (Hsp90 in cancer) are unrelated to the study. Many references are >10 years old; more recent literature on biphenyl antimicrobials and Suzuki coupling advances is omitted.
    Scheme 1: Poorly formatted with cramped text, unclear substitution patterns, and illegible atom labels.

*Answer: thank you for your suggestions and we correct the references and scheme as your comment*

11. Inconsistent Controls: Fluconazole is used as a control for C. albicans but not mentioned in methods. Streptomycin is used inconsistently across assays.
    Missing DLS/Polydispersity: For nanoparticle-like properties, DLS data would be relevant but not included. Terminology: "Gram-negative fungus strain Candida albicans" is incorrect—Candida is a yeast (fungus), not classified as Gram-negative.
    Units: Inconsistent use of mg/mL vs. μg/mL. Standard is μg/mL or μM.

***Answer:*** *Thank you for these important comments. We agree that controls, terminology, and unit reporting must be consistent for reproducibility. The manuscript has been revised accordingly:*

*(1) Controls (fluconazole/streptomycin)*

*We have now clearly described all positive and negative controls in the Methods section and standardized their use across assays.*

*(2) DLS/Polydispersity*

*We agree that DLS is relevant for nanoparticle or colloidal formulations. However, the present work investigates small-molecule biphenyl derivatives, not nanoparticle formulations; therefore, DLS/polydispersity analysis is not applicable to the study design.*

*(3) Terminology*

*We corrected the incorrect phrase “Gram-negative fungus strain.” Candida albicans is now correctly described as a yeast (fungus) throughout the manuscript.*

*(4) Units*

*We agree and have standardized all MIC and concentration units across the manuscript. MIC values and concentrations are now consistently reported in µg/mL*

**Answer comments reviewer 2:**

We sincerely thank Reviewer 2 for the detailed and constructive comments. We have carefully revised the manuscript to improve scientific rigor, consistency, clarity, and data presentation. Below we respond point-by-point.

1. Title
   The title should be revised to reflect the experimental scope.
   Currently, the title promises a lot of things without in-vivo experiments and mechanistic validation.

***Answer:*** *Thank you for this comment. We agree that the previous title could imply broader therapeutic and mechanistic conclusions than supported by the present study, which is limited to in vitro antimicrobial/anti-biofilm evaluation with supportive in silico analysis and no in vivo validation. Therefore, we revised the title to accurately reflect the experimental scope and to avoid overstatement.*

2. Keywords
   The keywords should not repeat terms already present in the title.
   More specific and relevant keywords should be included.

*Answer: thank you for your comment, we change the keywords according your suggest*

3. Introduction:
   • It requires language and grammatical revision to improve readability and clarity.
   • The rationale for selecting E. coli and C. albicans are not mentioned clearly and justified.
   • Recent literature on synthesis of biphenyl-based antimicrobials and antibiofilm agents should be incorporated.
   • The novelty should be clearly stated and the difference of the synthesized compound with the existing ones should be mentioned clearly.
   • The rationale behind selecting E. coli FabH as the docking target should be more clearly explained.

***Answer:*** *Thank you for these constructive comments. We have substantially revised the Introduction to improve language quality, clarity, and scientific justification. Specifically:*

*Language/grammar: The Introduction was rewritten to improve readability and scientific English, and repeated/awkward phrasing was removed.*

*Rationale for organism selection: We added clear justification for selecting E. coli (clinically relevant Gram-negative pathogen with permeability barrier) and C. albicans (clinically important yeast with strong biofilm-forming capacity).*

*Recent literature: We incorporated recent literature on biphenyl/biaryl antimicrobial and antibiofilm agents and added relevant citations.*

*Novelty: We explicitly stated the novelty of this work, emphasizing the focused substituted biphenyl series, systematic substituent variation, and identification of an active lead (3i) through combined antimicrobial/anti-biofilm screening.*

*FabH docking rationale: We expanded the rationale for selecting E. coli FabH as the antibacterial docking target (essential FAS-II enzyme, druggable active site, availability of co-crystal structure), and clarified that FabH docking is intended to support antibacterial interpretation only (not antifungal mechanism).*

4. Materials and Methods:

• This section requires major language correction and restructuring.
• The term gram–negative fungus is scientifically incorrect and must be corrected.
• CFU/ml values should be mentioned using standard scientific notation.
• Concentration used in the agar well diffusion metho should be mentioned clearly.
• Valid references should be cited for the standard protocols such as MIC.
• The rationale behind supplementing 1% xylose in the biofilm assay should be justified and supported by references.
• The differences between agar diffusion assays and broth-based MIC assays should be clarified and explained.

***Answer:*** *Thank you for these valuable comments. We have substantially revised and restructured the Materials and Methods section to improve clarity, grammar, and reproducibility. Specifically:*

*Language and structure: The entire section was rewritten with clear subheadings and consistent scientific English.*

*Terminology correction: The incorrect term “Gram-negative fungus” was removed. Candida albicans is now correctly described as a yeast (fungus) throughout the manuscript.*

*CFU/mL notation: Inoculum concentrations are now reported using standard scientific notation.*

*Standard MIC references: MIC determination is now described as broth microdilution, and appropriate standard references were added, along with a validated reference for resazurin-based microdilution.*

*1% xylose rationale: We clarified that 1% xylose was used as a standardized carbohydrate supplement to support consistent biofilm formation across replicates (applied equally to all groups) and added a published reference reporting TSB + 1% xylose in microtiter biofilm assays.*

5. Table
   • Tables require consistent units, formatting, and clear headings.
   • Computational predictions should be clearly labelled as in silico estimation.

Diffusion vs broth MIC: We explicitly clarified that agar diffusion assays provide screening outcomes influenced by diffusion/solubility, whereas broth microdilution is the reference method for MIC determination.

***Answer:*** *thank you for your comment, we change the keywords according your suggest*

6. Results and Discussion
   • Results should be presented more systematically with consistent units across all sections.
   • Molecular docking results should be interpreted as supportive evidence.
   • The discussion should integrate results with existing literature and explain similarities and differences with previous literature.
   • Statistical analysis should be clearly stated.

***Answer:*** *thank you for your comments, we do all the necessary according your feedback*
