# Supplementary material for: Synthesis of substituted biphenyls and in vitro evaluation of antimicrobial and anti-biofilm activities supported by in silico ADMET prediction
Source: PLoS One. 2026 Apr 6;21(4):e0346142. doi: 10.1371/journal.pone.0346142 (PMC13052900; doi:10.1371/journal.pone.0346142)
Supplement: Attachment — Submitted filename: Plos One Second Revision_29_1_26.docx [file pone.0346142.s004.docx]

**Manuscript ID: PONE-D-25-65844**

**REVIEW COMMENTS**

The manuscript is focused on the synthesis of substituted biphenyl derivatives and the evaluation of their antimicrobial, antibiofilm and in silico pharmacological properties. The topic is relevant and the experiments have been designed properly. However, the manuscript requires minor revision due to lack of clarity, consistency and overinterpretation of results with limited integration of previous literature.

Therefore, the manuscript requires minor corrections to improve the quality of the study.

1. Italicize scientific names (Staphylococcus aureus & E. coli).
2. Reduce the content in Materials and Methods and give precise protocol.
3. Typographical errors to be avoided throughout the manuscript.
4. Under Biological assays, the subheadings are to be modified.
5. DMSO to be mentioned as vehicle control (not negative control).
6. The concentration range is to be mentioned clearly in the methodology.
7. Delete “then” throughout the manuscript.
8. What was the diameter of the cork borer used?
9. Do not use the term “MIC-agar”; it can be mentioned as diffusion based screening for highest inhibitory concentration.
10. What is the rationale behind selecting xylose?
11. Inoculum size must be mentioned.
12. The authors are supposed to check for a couple of protein targets rather than going ahead with a single protein.
13. The authors are also requested to include the cytotoxic assessment, as it is essential to know its activity against normal flora or add a note about it.
14. NZ should be abbreviated.

**THE RESPONSE HAS TO BE GIVEN SPECIFICALLY FOR EACH COOMENT DO NOT PASTE THE SAME RESPONSE FOR MULTIPLE QUERIES.**
