# Supplementary material for: Synthesis of substituted biphenyls and in vitro evaluation of antimicrobial and anti-biofilm activities supported by in silico ADMET prediction
Source: PLoS One. 2026 Apr 6;21(4):e0346142. doi: 10.1371/journal.pone.0346142 (PMC13052900; doi:10.1371/journal.pone.0346142)
Supplement: Attachment — Submitted filename: Response to Reviewer.docx [file pone.0346142.s005.docx]

**Answer comments reviewer:**

We sincerely thank Reviewer for the detailed and constructive comments. We have carefully revised the manuscript to improve scientific rigor, consistency, clarity, and data presentation. Below we respond point-by-point.

1. Italicize scientific names (Staphylococcus aureus & E. coli).

*Answer: We agree. All scientific names of microorganisms were revised to italic formatting throughout the manuscript*

2. Reduce the content in Materials and Methods and give precise protocol.

*Answer: Thank you for these detailed methodological comments. The Materials and Methods section was shortened and restructured into concise subsections*

3. Typographical errors to be avoided throughout the manuscript.

*Answer: Thank you for your comments. The manuscript was carefully proofread and edited to correct typographical errors, spelling, punctuation, and consistency issues (units, abbreviations, and tense).*

4. Under Biological assays, the subheadings are to be modified.

*Answer: Thank you for your suggest. Long subheadings were replaced with concise, standard headings consistent with journal style.*

5. DMSO to be mentioned as vehicle control (not negative control).

*Answer: Thank you for raising this important issue. DMSO is now described as the vehicle (solvent) control, and we clarified that the final DMSO concentration was held constant across treated and control groups.*

6. The concentration range is to be mentioned clearly in the methodology.

*Answer: Thank you for these important comments. We explicitly reported the full tested concentration ranges and dilution schemes for both diffusion screening and broth microdilution MIC testing.*

7. Delete “then” throughout the manuscript.

*Answer: We agree. The word “then” was removed or replaced with more formal scientific phrasing throughout the manuscript.*

*Change made: Global edit applied.*

8. What was the diameter of the cork borer used?

*Answer: A 6 mm cork borer was used to punch wells in agar plates.*

9. Do not use the term “MIC-agar”; mention diffusion-based screening for highest inhibitory concentration.

*Answer: Thank you for highlighting this issue. The term “MIC-agar” was removed and replaced with diffusion-based screening terminology.*

10. What is the rationale behind selecting xylose?

*Answer: We clarified that 1% (w/v) xylose was used as a defined carbohydrate supplement to support consistent and reproducible biofilm formation under static microtiter conditions, applied equally to all treated and control wells to minimize nutrient-related variability. Appropriate literature support was added.*

11. Inoculum size must be mentioned.

*Answer: We agree. We added both the inoculum volume and density used in each assay. For the agar diffusion assay, 1.0 mL inoculum adjusted to 1.8 × 10⁸ CFU/mL was used.*

12. Check a couple of protein targets rather than a single protein.

*Answer: Thank you for this comment. We agree that multiple targets can provide stronger mechanistic insight. In this manuscript, docking was included as supportive antibacterial analysis and was limited to FabH due to study scope. We revised the text to avoid overinterpretation and added a limitation noting that future work will include additional validated antibacterial targets*

13. Include cytotoxic assessment or add a note about it (normal flora).

*Answer: We agree by this limitation. Cytotoxicity/selectivity testing was not performed within the scope of this study, and this is now clearly stated as a limitation. We added a future-work plan to evaluate cytotoxicity in non-cancerous mammalian cell lines and to calculate selectivity indices prior to any therapeutic claims.*

14. NZ should be abbreviated.

***Answer:*** *Thank you your note, we added NZ abbreviate*.
