# Supplementary material for: Synthesis of substituted biphenyls and in vitro evaluation of antimicrobial and anti-biofilm activities supported by in silico ADMET prediction
Source: PLoS One. 2026 Apr 6;21(4):e0346142. doi: 10.1371/journal.pone.0346142 (PMC13052900; doi:10.1371/journal.pone.0346142)
Supplement: Attachment — Submitted filename: Response_to_Reviewer_auresp_4.docx [file pone.0346142.s006.docx]

**Dear Editor,**

Thank you for the opportunity to revise our manuscript entitled "Synthesis of Substituted Biphenyls and In Vitro Evaluation of Antimicrobial and Anti-Biofilm Activities Supported by In Silico ADMET Prediction" (Manuscript ID: PONE-D-25-65844R2). We appreciate the thoughtful and constructive comments from the Editor, which have significantly improved the quality of our work.

**1. Chemical characterization**

For compounds 3a–3d, additional purity assessment should be provided. These compounds appear to be newly synthesized, yet only NMR and LC-MS data are reported. For newly reported compounds used in biological assays, it is generally expected to include at least one additional method confirming purity and molecular identity, such as HRMS or elemental analysis data.

*Answer: "We thank the reviewer for this valuable suggestion. In accordance with the journal's requirements for newly synthesized compounds, we had performed CHN analysis for all compounds 3a–3d.*

**2. Language and editorial revision**

The manuscript still contains numerous grammatical errors, tense inconsistencies, and awkward phrasing that affect readability. Examples include:

- “Suzuki coupling reactions were used to synthesis of biphenyl compounds…”
- “software were used to predicte the 3i compound pharmacokinetic properties.”
- “softwers were used to calculate the physiochemical properties.”
- duplicated wording in the methods section (e.g., “mixed gently, and allowed to solidify, mixed gently, and allowed to solidify”).

I recommend that the manuscript undergo thorough language editing before resubmission. The authors may consider using professional language-editing services or grammar-checking tools such as Grammarly, Write full, or similar writing software to improve grammar, syntax, and clarity.

*Answer: We thank the reviewer for this valuable recommendation. We agree that improving the language quality is essential for the clarity of our manuscript. For this reason, we used the Grammarly program to edit the manuscript according to your suggestion*

**3. Consistency in compound characterization**

The manuscript states that the compounds were characterized using IR and NMR techniques; however, IR data are not reported in the experimental section. Please clarify this point or include the relevant spectroscopic data.

*Answer: We thank the reviewer for pointing out this omission. The IR and CHN data were reported in the experimental section*
